# Design of a novel multi-epitope vaccine against Marburg virus using immunoinformatics studies

**DOI:** 10.1186/s12896-024-00873-2

**Published:** 2024-07-05

**Authors:** Fouad Qasim Jubair Al-Zayadi, Ali S. Shakir, Ahmed Shayaa Kareem, Abdolmajid Ghasemian, Esmaeil Behmard

**Affiliations:** 1 Department of Biology, College of Education for Pure Sciences, Al-Muthanna University, Al-Muthanna, Iraq; 2 College of Dentistry, University of Al-Qadisiyah, Diwaniyah, Iraq; 3 Department of Medical Laboratories Techniques, Imam Ja’afar Al-Sadiq University, Al-Muthanna, Babylon, 66002 Iraq; 4 Noncommunicable Diseases Research Center, Fasa University of Medical Sciences, Fasa, Iran; 5 School of Advanced Technologies in Medicine, Fasa University of Medical Sciences, Fasa, Iran

**Keywords:** Marburg virus, Multi-epitope vaccine, Prevention, immunization, In silico

## Abstract

Marburg virus (MARV) is a highly contagious and virulent agent belonging to *Filoviridae* family. MARV causes severe hemorrhagic fever in humans and non-human primates. Owing to its highly virulent nature, preventive approaches are promising for its control. There is currently no approved drug or vaccine against MARV, and management mainly involves supportive care to treat symptoms and prevent complications. Our aim was to design a novel multi-epitope vaccine (MEV) against MARV using immunoinformatics studies. In this study, various proteins (VP35, VP40 and glycoprotein precursor) were used and potential epitopes were selected. CTL and HTL epitopes covered 79.44% and 70.55% of the global population, respectively. The designed MEV construct was stable and expressed in *Escherichia coli (E. coli)* host. The physicochemical properties were also acceptable. MARV MEV candidate could predict comprehensive immune responses such as those of humoral and cellular in silico. Additionally, efficient interaction to toll-like receptor 3 (TLR3) and its agonist (β-defensin) was predicted. There is a need for validation of these results using further in vitro and in vivo studies.

**Supplementary Information:**

The online version contains supplementary material available at 10.1186/s12896-024-00873-2.

## Introduction

The Marburg virus (MARV) is a highly contagious and virulent agent that belongs to the *Filoviridae* family and develops severe hemorrhagic fever in humans and non-human primates [1]. The virus is currently concentrated in sub-Saharan Africa and sporadically to other regions [2] and is transmitted through contact with the bodily fluids of infected animals or humans and has a range of high fatality rate [3–5]. Symptoms appear within 2–21 days following exposure and the incubation period commonly includes 5–10 days [6]. There is currently no approved drug or vaccine for MARV, and management mainly involves supportive care to heal symptoms and prevent complications [7–9]. These symptoms may include high fever, myalgia, headache and fatigue, chills and sweating, nausea and vomiting, abdominal pain and diarrhea [8, 9]. As the disease progresses, more severe symptoms may appear, such as skin rash, internal and external bleeding, shock and multiple organ failure [4, 10, 11]. Notably, the disease and complications may be more severe among certain groups [8, 12] such as the immunosuppressed and elderly individuals, pregnant women and children [13]. It has been classified as Category A bioterrorism agent by the US Centers for Disease Control and Prevention (CDC).

MARV contains several virulence factors. The envelope glycoproteins (GPs) include primary viral surface proteins mediating attachment to the cellular carbohydrates and penetration into host cells via membrane fusion [14]. The GP forms a trimeric spike on the surface of the virus, which interacts with cellular receptors on target cells and mediates membrane fusion, allowing the virus to enter the cell using macropinocytosis and vesicles [15–17]. Nucleoprotein (NP) is a structural protein encapsidating the viral genome and playing a critical role in the viral replication and assembly. It also interacts with host factors to modulate the host immune responses [18–21]. Viral protein 35 (VP35) is a multifunctional protein, acting as a suppressor of the host innate immune response by inhibiting the production of interferons, which are important antiviral cytokines. VP35 also plays a role in viral transcription and replication [22–24]. Viral protein 40 (VP40) is a matrix protein and have a key role in viral assembly and budding via interacting with other viral proteins and host factors and envelope formation [14, 25]. Viral protein 24 (VP24) is a multifunctional protein which inhibit the host interferons release by blocking the nuclear translocation of host transcription factors. It also interacts with other viral proteins to regulate viral replication and assembly [26, 27]. These proteins work together to enable MARV to evade the host immune system, replicate efficiently, and cause severe disease [14]. Following the infection, the host immune responses including innate and adaptive arms of B cells and T cells (CD4 + and CD8+) are activated [4, 28–30]. Multi-epitope vaccines (MEVs) have several advantages in terms of efficient immune responses, unwanted responses and low allergenicity and side effects [31, 32]. The aim of our study was to design a MEV candidate for MARV in silico.

## Methodology

### Protein sequences retrieval

The sequences of viral glycoprotein precursor (AAC40460.1), polymerase cofactor VP35 (P35259.1) and matrix protein VP40 (P35260.3) were obtained from NCBI (https://www.ncbi.nlm.nih.gov/) database. Then, the sequences of proteins were saved in FASTA format for subsequent analyses.

### Epitope prediction

#### MHC-I binding epitopes prediction

The elicitation of cytotoxic T-cells (CTLs) is pivotal for combating viral infections, and hence, prediction of specific epitopes is pivotal by MEV candidate design. The Immuno Epitope Database and Analyzing Resource (IEDB-AR) (www.iedb.org) is applied for T cells epitopes prediction mainly of major histocompatibility complex (MHC)-I and binding ligands. Accordingly, we used IEDB web server to predict epitopes with 11 mer length having the potential of binding to the MHC-I using the ANN 4.0 prediction method. Viral potential epitopes from glycoprotein precursor, VP35 and VP40 proteins were utilized to predict CTLs eliciting. The percentile rank < 1 and IC_50_ ≤ 50 nM were selected as the cut-off values for screening of high affinity epitopes and the MHC source species was selected as human.

### Helper T lymphocyte (HTL) epitopes prediction

The elicitation of helper T-cells (HTLs) is pivotal for combating viral infections and hence, prediction of specific epitopes is crucial for an MEV candidate design. Accordingly, we used IEDB web server (www.iedb.org) to predict epitopes with 15 mer length and potential of binding to the MHC-II using the NN-align 2.3 prediction method to predict HTL epitopes. The human HLA-DR was used in the MHC source species with higher antigenicity score and high affinity epitopes were screened by adjusting the rank < 1 as the cut off value.

### The antigenicity, allergenicity, toxicity and population coverage assessment

The antigenicity of epitopes was predicted utilizing VaxiJen v2.0 webserver (http://www.ddg-pharmfac.net/vaxijen/VaxiJen/VaxiJen.html*)* (by set of 0.4 threshold). Their toxicity was also assessed by the employment of ToxinPred server. Moreover, epitopes allergenicity was checked using AllerTop (https://www.ddg-pharmfac.net/AllerTOP/*)* server. The population coverage analysis tool predicts the coverage of a vaccine candidate by determining the percentage of the population predicted for recognizing the selected epitopes based on MHC alleles (http://tools.iedb.org/population/). Based on this information, the potential efficacy of a vaccine in diverse populations is predicted. By using the CTL and HTL epitopes individually and in combination, the assessment of the vaccine candidate coverage across various populations is evaluated. Additionally, by emphasizing the total coverage of selected alleles across multiple continents, insights into the global reach of the vaccine candidate is achieved.

### The MEV characterization and structural mapping

MARV glycoprotein precursor, VP35 and VP40 proteins high priority epitopes were screened and included into the MEV construct. Additionally, toll-like receptor 3 (TLR3) agonist (β-defensin) was fused to epitopes sequences for the MEV candidate construct designing. The AAY (CTL epitopes), GPGPG (HTL epitopes) KK (B-cell epitopes) and EAAAK (adjuvant) linker sequences were utilized for binding of MEV various segments. Subsequently, Expasy’s ProtParam online server (http://web.expasy.org/protparam/*)* was employed for the evaluation of MEV physicochemical features including grand average of hydropathicity (GRAVY), molecular weight, number of positive and negative residues, isoelectric point (pI), number of amino acids, and aliphatic and instability indices. In addition, SOLpro online server (http://scratch.proteomics.ics.uci.edu*)* was applied for its structural solubility within *E. coli*. Furthermore, MEV antigenicity and allergenicity were predicted using associated aforementioned servers.

### Three-dimensional structure homology modeling and refinement

The MEV three-dimensional (3D) structure was predicted using online AlphaFold2 tool. Afterwards, the 3D model structure was relaxed utilizing Galaxy Refine web server through repacking and the MD simulation. Furthermore, the MEV candidate quality assessment was conducted using ERRAT (http://services.mbi.ucla.edu/ERRAT/), SWISS-MODEL (https://swissmodel.expasy.org/) and ProSA web servers. The prediction of MEV 3-D structure linear and discontinuous B-cell epitopes was performed using the Ellipro in the IEDB database (http://tools.iedb.org/ellipro/*)* [33].

### Molecular docking of multi-epitope vaccine-toll-like receptor 3 interactions

The MEV-TLR3 interactions are crucial for eliciting efficient immune responses. Accordingly, the TLR3 3D structure (PDB ID: 2A0Z) was extracted from the protein data bank (www.rcsb.org*)* and was docked with the MEV candidate using ZDOCK server (http://zdock.umassmed.edu*)* to uncover the binding patterns [34].

### Molecular dynamics simulation of the vaccine-TLR3 complex

The MD simulation for all atoms was performed for the analysis of dynamical behavior of MEV-TLR3 docked complex and its structural stability using GROMACS package 2022.6 [35]. Primarily, the force field included OPLS-AA [36] and explicit solvent included three-site (TIP3P) model water molecule for the solvation of the complex system [37]. Additionally, chloride and sodium ions at optimal concentration were added to mimic natural or physiological conditions. Periodic bounding conditions were considered in the simulation box [35]. Moreover, the long-range electrostatics was assessed utilizing particle mesh Ewald (PME) [38]. The LINCS algorithm was applied for restraining the bonds length with a cut-off of 1.2 nm for Coulombic bonds and van der Waals connections [35]. To minimize the energy and refine unsuitable contact of the geometry, the steepest descent algorithm was applied. In the NVT ensemble, the system’s temperature was gradually increased to reach 310 K during 0.5 ns and in the NPT ensemble the pressure and density were adjusted at 1 atm pressure for 0.5 ns. Then, the main simulation on protein molecules with no restrain was done for 100 ns and the energy analysis of binding profile during MD simulation was implemented using the MMPBSA method and the structure was visualized using the open-source molecular visualization (PyMOL) software [39].

### Immune simulations

The C-ImmSim server version 10.1 was employed to simulate the immune responses against the MEV construct. Accordingly, the lymph nodes, thymus and bone marrow were simulated and the incorporated parameters included HLA (DRB1_0101, A0101 and B0702 each in pair) for 100 steps and one injection, 10 volumes and 12,345 random seeds [40].

### The multi-epitope vaccine codon optimization and in silico cloning

The codon optimization method enhances the expression efficiency of foreign genes within a host organism. The Java Codon Adaptation Tool (JCat) was utilized for back translation, codon optimization, and determination of the codon adaptation index (CAI) value and GC content of the vaccine sequence [41]. The protein sequence of the designed MEV was subjected to the JCat server, with *E. coli* (strain K12) as the host organism. Specific options were used to prevent transcription termination, enhance ribosome binding, and avoid restriction enzyme cleavage sites. HindIII and HpaI recognition sequences were also incorporated at the N- and C-terminal ends of the vaccine sequence. The final optimized sequence was then inserted into the pET28a (+) vector using SnapGene 3.2.1 software.

## Results

### T cell and B cell epitopes prediction

The elicitation of durable and multi-functional immunity against infections, particularly via CTL and HTL epitopes is promising by designing an efficient vaccine candidate. Accordingly, CD8^+^ cells combating intracellular pathogens are provoked via CTL epitopes, while CD4^+^ cells cooperate with humoral and cellular immunity and are provoked via HTL epitopes. Moreover, B cells participate in humoral responses to infectious agents. Considering these, potential specific epitopes selection for CTL and HTL subtypes and B cells is crucial in a vaccine designing process. Those potential CTL, HTL and B cell epitopes have been inferred in Tables 1, 2 and 3 and Supplementary Data S1-S6.

**Table 1:** The final CTL epitopes for the MEV construction

Final CTL Epitopes	Protein	Antigenicity	Allergenicity
^56^SIHPNLPPIVL^66^	VP40	1.16	non-allergen
^32^RQLHEITPVLK^42^	VP35	0.71	non-allergen
^10^QTVPRPSQKSL^20^	VP35	0.62	non-allergen
^58^KVADSPLEASK^68^	GP	0.67	non-allergen
^8^RIASTTMYRGR^18^	GP	0.58	non-allergen

**Table 2:** The final HTL epitopes for the MEV construction

Allele	Final HTL Epitopes	Protein	Antigenicity	Allergenicity
HLA-DRB1*15:01, HLA-DRB3*02:02, HLA-DPA1*02:01/DPB1*05:01, HLA-DQA1*01:02/DQB1*06:02, HLA-DPA1*02:01/DPB1*01:01, HLA-DPA1*03:01/DPB1*04:02, HLA-DRB1*04:05, HLA-DRB1*12:01, HLA-DPA1*01:03/DPB1*04:01	^1^MASSSNYNTYMQYLNPPPYADHGANQLIPADQLSNQQGITPNYVGDLNLDDQFKGNVCHA^60^	VP40	0.44	non-allergen
HLA-DRB1*11:01, HLA-DRB4*01:01,HLA-DQA1*01:02/DQB1*06:02,HLA-DQA1*01:01/DQB1*05:01, HLA-DRB1*09:01, HLA-DRB5*01:01, HLA-DRB1*01:01, HLA-DRB1*08:02	^2^WDSSYMQQVSEGLMTGKVPIDQVFGANPLEKLYKRRKPKGTVGLQCSPCLMSKAT^56^	VP35	0.44	non-allergen
HLA-DQA1*03:01/DQB1*03:02, HLA-DRB1*12:01, HLA-DRB3*02:02, HLA-DRB1*13:02, HLA-DQA1*05:01/DQB1*02:01, HLA-DRB1*04:05, HLA-DRB1*04:01, HLA-DQA1*03:01/DQB1*03:02, HLA-DRB1*04:05	^1^MKTTCLFISLILIQGIKTLPILEIASNNQPQNVDSVCSGTLQKTEDVHLMGFTLSGQKVADSPLE^65^	GP	0.73	non-allergen

**Table 3:** The final B-cell epitopes for the MEV construction

Sequence	Protein	Start position	Score	Antigenicity	Allergenicity
VGLQCSPCLMSKATST	VP35	43	0.92	1.04	NON-ALLERGEN
MASSSNYNTYMQYLNP	VP40	1	0.95	0.52	NON-ALLERGEN
IIDISAYNERTVKGVP	VP40	66	0.93	0.91	NON-ALLERGEN
TVKKQAYRQHKNPNNG	VP40	208	0.91	0.50	NON-ALLERGEN
DGLINAPIDFDPVPNT	GP	449	0.98	0.64	NON-ALLERGEN
GPGIEGLYTAGLIKNQ	GP	537	0.91	0.45	NON-ALLERGEN
QGYRHMNLTSTNKYWT	GP	184	0.90	1.02	NON-ALLERGEN
QGIKTLPILEIASNNQ	GP	14	0.90	0.57	NON-ALLERGEN

Potential antigenic, non-toxic and immunogenic epitopes were used and evaluated for their affinity to MHC-I and MHC-II alleles. Then, the MEV 3D structure was modeled and refined. For population coverage analysis, the IEDB population coverage analysis tool was utilized to predict the population coverage of specific CTL and HTL epitopes in the MEV. The analysis revealed that CTL and HTL epitopes covered 79.44% and 70.55% of the global population, respectively. When used in combination, the population coverage of CTL and HTL epitopes reached 93.94%. In terms of regional population coverage, Europe had the highest coverage at 96.81%, followed by North America at 94.66%, East Asia at 91.38%, Northeast Asia at 87.62%, West Indies at 85.91%, South Asia at 84.31%, Southeast Asia at 83.47%, Oceania at 81.75%, North Africa at 80.15%, South America at 78.44%, West Africa at 76.74%, Southwest Asia at 73.2%, East Africa at 72.94%, Central Africa at 72.21%, South Africa at 51.75% and Central America at 46.71%.

### Multi‑epitope vaccine structural characterization

**Fig. 1:**
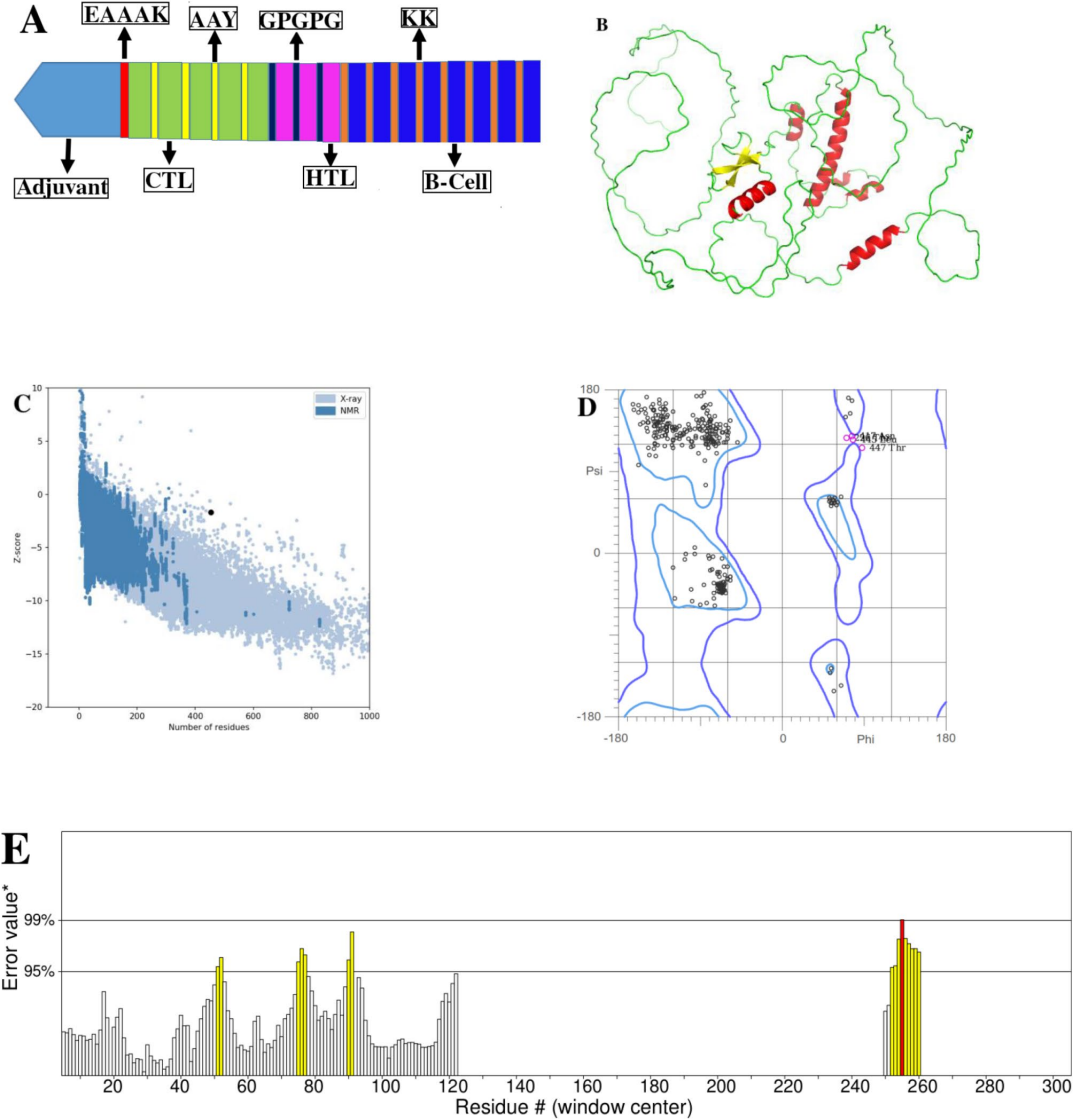
(**A**) Schematic profile of the MEV candidate with 588 residues length. A CTB as an adjuvant was attached to the N-terminal region of the MEV using EAAAK linker, followed by 5 CTL, 3 HTL and 8 B-cell epitopes integrated by AAY, GPGPG and KK linkers; (**B**) 3D structure model of the MEV protein; (**C**) Ramachandran plot assessment of refined structure; (**D**) ProSA value of 3D MEV model showing Z-score (-1.68); (**E**) The ERRAT overall quality factor of 3D MEV model

High potential epitopes with predetermined conditions were selected from protein sequences and incorporated into MEV construct (Supplementary Table S1 and S2, Supplementary data S1-S6) and the overlapping regions containing HTL epitopes were taken from the protein sequences. Therefore, the MARV MEV construct in linear form contained five CTL, three HTL and eight B cell epitopes (Tables 1 and 2) fused via AAY, GPGPG and KK linkers respectively. Moreover, the TLR3 agonist (β-defensin adjuvant) was attached to the N- terminal region via EAAAK linker (Fig. 1A). The MEV molecular weight included 49.742 kDa having 454 amino acids. Next, its 3D structure was modeled [42] and refined (Fig. 1B). The best model for structural quality was selected with a Z-score of -1.68 (Fig. 1C), which was in range of comparable size proteins, being reliable (Fig. 1C). In Ramachandran plot (Fig. 1D), 95.58% of residues were in favored region, 2.13% in allowed and 2.29% in outlier regions of the 3D-model structure, confirming its overall quality. As the cut-off reliability is considered ≥ 90% for residues in the favoured region [43], the current MEV residues were accordingly located in the favoured region, confirming its reliability. The ERRAT overall quality factor included 87.4 (Fig. 1E), indicating acceptable quality and validity percentage (Fig. 1E), considering ERRAT score cut-off of 50 as a good structural quality [44].

### Immunogenicity, allergenicity and physicochemical characteristics

The MEV candidate physiochemical traits and antigenicity, allergenicity, safety and solubility unveiled a valid and appropriate designation (Table 4). The MEV aliphatic index (73.30, indicating thermo-stability), theoretical pI (9.70) and half-life in mammalian reticulocytes, yeast and *E. coli* included > 30 h, > 20 h and > 10 h, respectively. The GRAVY score included − 0.56 exhibiting its hydrophilic nature which facilitates its interaction with other proteins. Moreover, its solubility upon over-expression included 0.71 in aqueous environment confirming the MEV solubility and its instability index was 38.57 indicating its high stability.

**Table 4:** The MEV physiochemical traits, antigenicity and allergenicity

Features	Assessment
Number of amino acids	454
Molecular weight	49742.41 Dalton
Theoretical pI	9.70
No. of negatively charged residues (Asp + Glu)	28
No. of positively charged residues (Arg + Lys)	63
Extinction coefficient (at 280 nm in H2O)	46,020 M^− 1^cm^− 1^
Instability index	38.57
Aliphatic index	73.30
Grand average of hydropathicity (GRAVY)	-0.56
Antigenicity	0.95 (AntigenPro), 0.57 (Vaxijen v.2.0)
Allergenicity	Probable non-allergen (AllergenFP v.1.0) Probable non-allergen (AllerTOP v.2.0)

### B cell epitopes prediction

The humoral immunity is provided by B cells which have substantial role with this regard and can develop memory immunity. Therefore, sufficient B cell receptors should be targeted by adding B cell epitopes in the MEV construct. The B cell continuous and discontinuous (linear and conformational, respectively) epitopes were predicted considering default parameters of Ellipro server. Accordingly, three continuous epitopes having scores of 0.95, 0.94 and 0.90 and two discontinuous epitopes with scores of 0.95 and 0.94 were obtained (Table 5). In addition, 94% of residues were in the predicted ellipsoid zone considering PI value of 0.94 which inferred their high solvent accessibility. The predicted epitopes (two conformational and three linear) demonstrated humoral immunity eliciting by the MEV.

**Table 5:**
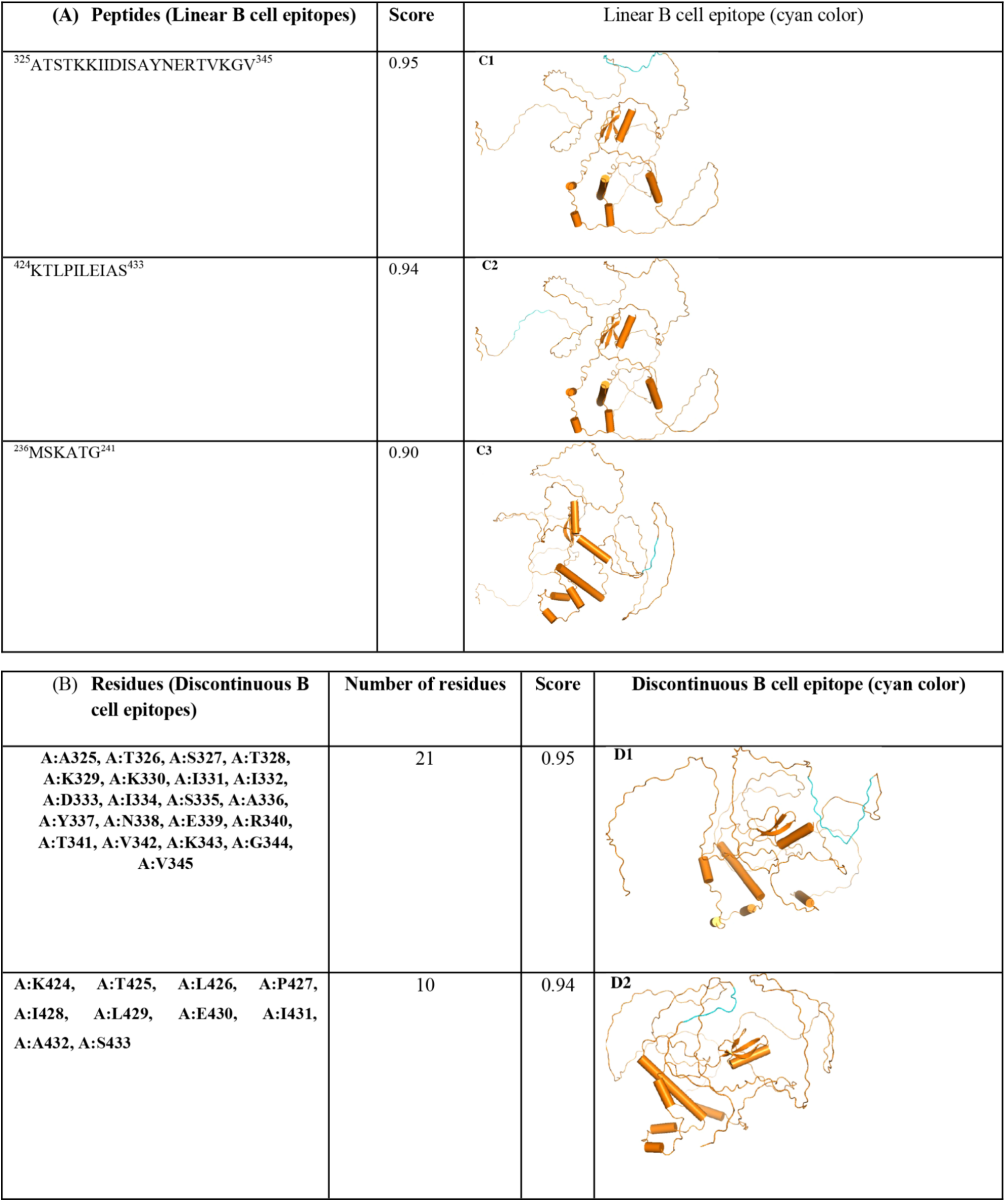
Linear (A) and discontinuous (B) B cell epitopes

### Molecular docking of the MEV-TLR3

TLRs are a part of native immunity which recognize the pathogens structures and also activate acquired immune responses. Viral envelope proteins bind to TLR1, TLR2, TLR3, TLR4, TLR6 and TLR10 and mediate the release of pro-inflammatory cytokines and activate immunity. TLR3 is expressed in mast cells (MCs), macrophages and myeloid DCs. For studying the stability of MEV and TLR3 agonist (β-defensin adjuvant) interactions, the docking was conducted (using the ZDOCK server). Accordingly, top-ranked scores of ligand poses are selected as potential binding modes. These interactions determine the durability of responses. Considering these conditions, the proper docked structure was taken. Accordingly, the optimal docked complex was considered for the running in the MD simulation studies.

### Molecular dynamic simulation

**Fig. 2:**
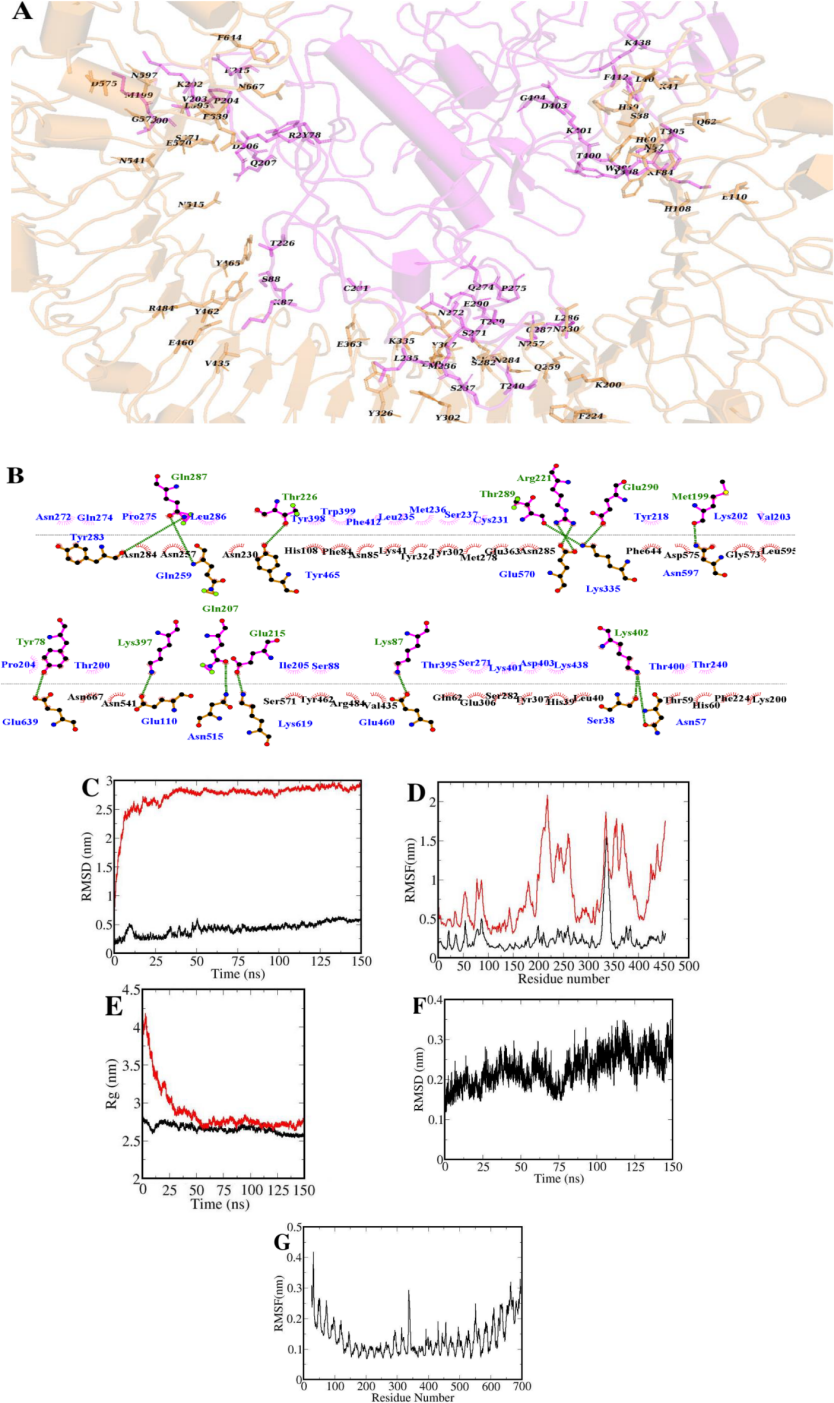
(**A**) MEV (magenta cartoon)-immune receptor (light brown cartoon) complex after 150 ns simulations time; (**B**) The interface residues related to MEV (magentas line) and (brown line) are labeled. Illustration of the molecular dynamic equilibration for simulation outputs; (**C**) Root mean squared deviations (RMSDs) of Cα for subunit MEV at free (red line) and complex (black line) states (**D**) Root mean squared fluctuations (RMSFs) of Cα atoms for subunit MEV at free (red line) and complex (black line) states. (**E**) Radius of gyration of the subunit MEV at free (red line) and complex (black line) states. (**F**) RMSDs of Cα for TLR3 at complex state. (**G**) RMSFs of Cα atoms for TLR3 at complex state

During the MD simulations, interactions between MEV and TLR3, their complex stability and conformational changes as well as efficient immune recognition of various epitopes were extrapolated. The MEV-TLR3 docked complex structural stability was assessed for 150 ns MD simulation trajectory (Fig. 2).

The RMSD scores of the MEV Cα atoms at free and complex states initially enhanced before 25 ns and then had smooth fluctuations outlining its structural stability (Fig. 2C). Furthermore, the MEV local structural flexibility was calculated using the root mean square of fluctuation (RMSF) scores. The RMSF plot of MEV inferred high flexibility in loop regions in the free form which was incredibly decreased in complex state with TLR3, due to various inter-molecular interactions (Fig. 2D). Additionally, radius of gyration (Rg) of the MEV which determines the structure compactness was calculated exhibiting an approximate mean Rg values of 2.9 nm and 2.6 nm for MEV at free and complex forms, respectively (Fig. 2E). The flexible regions alterations and conformational and regional movements are determined by Rg fluctuations to uncover the MEV characters for incorporation in the binding pocket in complex form. The TLR3 structural stability and flexibility during 150 ns of simulation time was demonstrated by evaluation of root mean square of deviation (RMSD) of Cα atoms (Fig. 2F). Its RMSD values indicated that the TLR3 had good structural stability throughout the simulation time. The RMSF values of the Cα atom in a TLR3 provide insight into the flexibility and dynamics of the protein (Fig. 2G). Higher RMSF values indicate greater flexibility and movement, while lower values suggest a more rigid structure. Regions of the TLR3 with high RMSF values are typically associated with loops, termini, or other flexible segments.

Analyzing the RMSD and RMSF trends provides insights into the dynamics of MEV and TLR3, as well as conformational changes in response to vaccine binding. Additionally, comparing the RMSD profiles of different protein simulations to simulated structures provides valuable information about the accuracy and reliability of our simulation results. MEV-TLR3 complex efficient interactions and binding stability was outlined at various time intervals by RMSF pattern which inferred low rate of structural or conformational changes in the flexible loop regions, indicating the stability.

### MEV-TLR3 free energy of the binding

The binding strength between MEV and TLR3 structures was determined by MMPBSA approach, in which the binding free energy was calculated between them. The polar and nonpolar energies (∆E_polar_ = -2513.13 ± 131.92 kJ/mol, ∆E_non−polar_ = -890.66 ± 43.66 kJ/mol) of components play a determining role in the MEV-TLR3 complex stability (Table 5). In addition, the component favorable electrostatic energy (ΔE_ele_ = -4525.13 ± 237.34 kJ/mol) is crucial for the TLR3 and MEV binding process. The primary driving force for binding of structures was polar term in which both hydrophilic and hydrophobic interactions determine thermodynamically favorable binding between the MEV and TLR3 (∆G_binding_ = -3403.79 ± 133.21 kJ/mol) (Table 6).

**Table 6:** Binding free energy values for the MEV-TLR3 complex

Interaction energy	TLR3-MEV
∆E_ele_^a^	-4525.13 ± 237.34
∆E_vdW_^b^	-775.88 ± 75.96
∆G_PB_^c^	2012.96 ± 176.24
∆G_SA_^d^	-114.78 ± 11.36
∆E_non−polar_^e^	-890.66 ± 43.66
∆E_polar_^f^	-2513.13 ± 131.92
∆G_bind_	-3403.79 ± 133.21

^a^ Electrostatic contribution, ^b^ van der Waals contribution, ^c^ Polar contribution of the solvation effect, ^d^ Non-polar contribution of solvation effect, ^e^ ∆E_polar_ = ∆E_ele_ + ∆G_GB_, ^f^ ∆E_non−polar_ = ∆E_vdW_ + ∆G_SA_

### Immune responses simulation

**Fig. 3:**
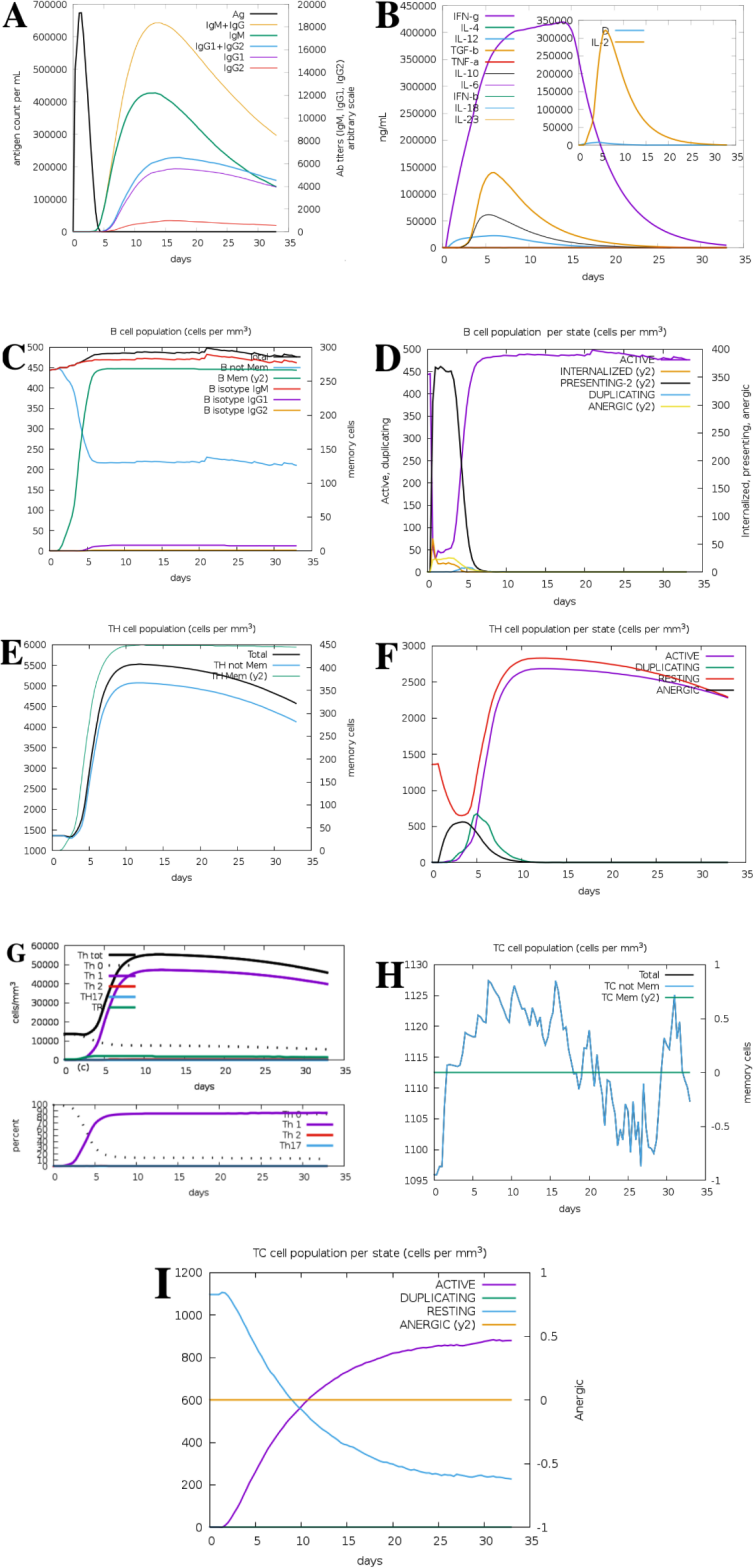
Immune responses to MARV including antibodies (**A**), cytokines (**B**), B cells (**C** and **D**) and T cells (**E**-**I**). Accordingly, the antibodies levels enhanced nearly two weeks after antigen exposure (**A**). Additionally, highest increase in cytokines levels included for IFN-b and TGF-b five to 15 days post-vaccination (**B**). The increase in the number of B cells including memory cells and IgM, IgG1 and IgG2 isotypes and active B cells was predicted after 2–3 days of vaccination (**C**, **D**). Moreover, T cells populations but not anergic cells enhanced following 2–3 days of vaccination. TH cells including total T_H_ and T_H_1 cells mostly increased 2–3 days after vaccination (**E**, **F**). Additionally, the enhanced percentage of T_H_1 cells continued for more than a month (**G**). Not memory T_C_ cells count increased 2 days after vaccination and maximized 7–17 days and then decreased to day 28 and increased again 28–31 days. Active T_C_ cells number increased following 2–3 days after vaccination and memory T_C_ cells had a stationary state during the simulation

The proper combating to MARV is exerted by both the innate and acquired immune responses. To assess this process, C-IMMSIM immune server was employed to determine the immunogenic profile of the MEV construct. Accordingly, initial release of the IgM, and IgM + IgG, IgG1 and IgG1 + IgG2 caused a rapid mitigation of antigen (Ag) (Fig. 3A). IgG responses are pivotal for the disease control. Additionally, various B cell isotypes (isotype switching) were observed indicating memory formation. Various cytokines and interleukins were also released (Fig. 3B). The increase in B cells population was in association to the IgG enhancement levels (Fig. 3C and D). As memory B cells and T cells (eliciting HTLs and CTLs responses) were developed, dramatic immunologic provocation was observed against the MEV with memory Tc and Th cells (Fig. 3E-I). INF-γ and IL-2 at high levels alongside T_H_1 and memory T cells also highlighted acceptable immune responses by activation of various cells such as B cells, Tc and Th cells, natural killer (NK) cells and macrophages. Therefore, the MEV candidate could provoke efficient and durable host responses against MARV.

**Fig. 4:**
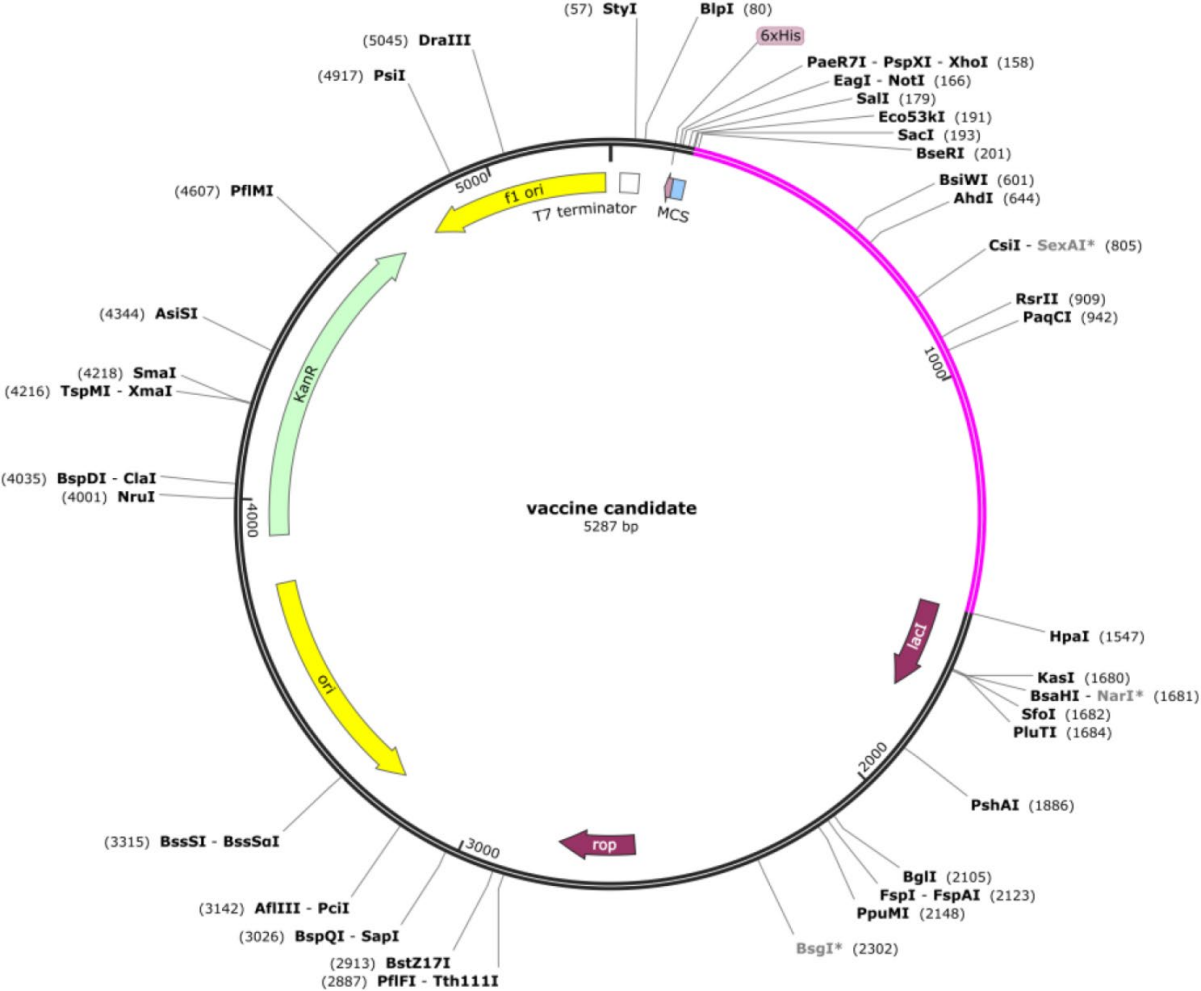
In silico cloning involved inserting the sequence of the MEV (highlighted in magenta) between HindIII (173) and HpaI (1547) sites in the pET-28a (+) expression vector (depicted in black)

### The multi-epitope vaccine codon optimization and in silico cloning

The multi-epitope vaccine’s protein sequence was reverse translated into a 1362 bp nucleotide sequence using the JCat server. The server predicted that the optimized sequence had a GC content of 50.73% and a CIA value of 1.0. Subsequently, the vaccine’s nucleotide sequence was cloned in silico into the multiple cloning site (MCS) of pET28a(+) between the HindIII (173) and HpaI (1547) restriction sites, producing a recombinant plasmid with a length of 5287 bp (Fig. 4).

## Discussion

Currently, there are no approved vaccines to control MARV infection for clinical application. However, several experimental vaccines are currently being developed and evaluated. One promising vaccine candidate is based on an attenuated form of the virus (Vesiculovax), which has been genetically modified to preclude infection and provoke immune responses [45]. Replication Incompetent Vaccines deliver genetic materials encoding MARV proteins for the elicitation of humoral responses. In vivo studies have been performed in cynomolgus macaques [1, 46]. Clinical trials of these vaccines [NCT00605514, NCT02661464] are also ongoing [6]. In addition, a vaccine developed by the Public Health Agency of Canada and NewLink Genetics, unraveled promising results in non-human primates and was evaluated in Phase 1 clinical trials at the time. Another vaccine candidate, developed by the Russian company Gamaleya Research Institute of Epidemiology and Microbiology, entered into Phase 1 clinical trials at the time [47]. Whole virus inactivated MARV with 50% survival stimulated the immune system to produce a protective response against the actual virus for 21 days of challenge in Rhesus monkeys [48]. Several experimental whole virus and inactivated or attenuated vaccines for MARV have been developed and evaluated in animal models, showing promising results in terms of inducing protective immunity against the virus. For example, one study found that an inactivated vaccine based on the Angola strain of MARV was able to protect non-human primates from lethal infection with the same strain of the virus [49, 50]. Virus-like Replicon Particle (VRP) using GP, NP and GP + VRP-MARV NP at three doses for each, respectively had survival rates of 100%, 67% and 100% after 35 days of challenge [51]. VLP modality using MARV GP, MARV GP + NP, MARV GP/NP/VP40 + Poly-IC and MARV GP/NP/VP40 + QS-21 in three doses after 28 days challenge had 100% survival rate in macaques [52].

In this study, the designed MARV MEV construct (molecular weight of 49.742 kDa with 454 amino acids) contained five CTL, three HTL and eight B cell epitopes in linear form. In Ramachandran plot, 95.58% of residues were in favored region, confirming its reliability. It also had a good structural quality. In addition, the MEV candidate physiochemical traits and antigenicity, allergenicity, safety and solubility were determined. Molecular docking of the MEV-TLR3 was conducted and the optimal docked complex was considered for the running in the MD simulation studies.

During the MD simulations, interactions between MEV and TLR3, their complex stability and conformational changes as well as efficient immune recognition of various epitopes were extrapolated. The MEV-TLR3 docked complex structural stability was assessed for 100 ns MD simulation trajectory. The structural stability of TLR3 and MEV was confirmed by RMSD evaluation. Furthermore, the MEV and TLR3 local structural flexibility was calculated using the RMSF scores. Regarding the B cell epitopes, two discontinuous epitopes with scores of 0.95 and 0.94 and three continuous epitopes having scores of 0.95, 0.94 and 0.90 were obtained. In addition, MEV-TLR3 free energy of the binding demonstrated their stable interactions considering polar and nonpolar energies and hydrophilic and hydrophobic interactions.

Viral glycoproteins have been utilized for MEV designing for MARV [53]. In a study, VP40 and envelop glycoproteins of MARV were used and a MEV was designed and docked with TLR8. The MEV was expressed in *E. coli* and elicited efficient immune responses in silico [54]. In another study, MARV structural proteins including GP, L, VP24, VP30, VP35 and VP40 were used to design a MEV and could elicit B and T cells responses in silico. It had also high affinity to TLR4 [55]. MARV surface GP and fusion proteins employed for a MEV designing, was docked with TLR2, TLR4, and TLR5 and could provoke robust and sustained immune responses [56].

MEVs containing multiple epitopes or antigenic determinants from target pathogen are promising in vaccine development with various advantages [55]. In the case of the MARV, various epitopes have been used from virus components, such as its surface glycoproteins, nucleocapsid proteins, and matrix proteins [55]. One advantage of an MEV is capability of provoking a broad immune response against various components of the pathogen, potentially providing greater protection compared to single-epitope vaccines [31, 57]. Additionally, MEVs can be designed to target conserved regions of the pathogen, reducing the risk of vaccine escape mutants. Several studies have assessed MEVs against viral pathogens [31, 58]. A MEV was designed for severe acute respiratory syndrome coronavirus 2, selected spike glycoprotein and nucleocapsid proteins and tetanus toxin fragment C (TTFrC) and cholera toxin b (CTB) adjuvant which was docked to TLR-2 and TLR-4. It was structurally proper and could elicit humoral and cellular responses [59]. Another MEV against *Proteus penneri*, containing four potential epitopes, was docked to TLR-4 and could elicit sufficient immune responses [60]. In another MEV construct designed using hepatitis C virus E2 protein conserved regions, CTB was selected as the adjuvant and the construct was docked to TLR2 and TLR4 which provoked B and T cells responses [61]. Using epitopes derived from the glycoprotein, nucleoprotein, and matrix protein (GP, VP35, VP24, VP30, VP40) of MARV, in vitro experiments unraveled that the vaccine was capable of inducing a strong antibody response against the virus and high affinity to TLR4. Overall, while more research is needed, MEVs hold promise as a potential strategy for developing effective vaccines against MARV [55]. Main limitations of this study included lack of experimental evaluation of MEV-mediated responses.

## Conclusion

MARV is a highly virulent viral pathogen causing severe hemorrhagic fever in humans and non-human primates. The virus is classified as a biosafety level 4 (BSL-4) pathogen, which means that it requires the highest level of containment protocols to prevent accidental exposure and spread. Currently, there is no approved vaccine or specific treatment for the virus. Our results inferred that the MEV could provoke immune responses of B cells and T cells in silico. The MEV construct was stable and expressed in *E. coli* host. There is a need for validation of these results using further in vitro and in vivo studies.

### Electronic supplementary material

Below is the link to the electronic supplementary material.

Supplementary Material 1

Supplementary Material 2

Supplementary Material 3

Supplementary Material 4

Supplementary Material 5

Supplementary Material 6

## Data Availability

No datasets were generated or analysed during the current study.
